# Molecular surveillance for dengue serotypes among the population living in Moyen-Ogooué province, Gabon; evidence of the presence of dengue serotype 1

**DOI:** 10.1186/s12985-024-02406-x

**Published:** 2024-06-20

**Authors:** Rodrigue Bikangui, Soulemane Parkouda, Ayong More, Marien Veraldy Magossou Mbadinga, Ismael Piérrick Mikelet Boussoukou, Georgelin Nguema Ondo, Anne Marie Mouina Nkoma, Rafiou Adamou, Yabo Josiane Honkpehedji, Elie Gide Rossatanga, Yuri Ushijima, Haruka Abe, Bertrand Lell, Jean Claude Dejon-Agobé, Jiro Yasuda, Ayola Akim Adegnika

**Affiliations:** 1 Centre de Recherches Médicales de Lambaréné (CERMEL), Lambaréné, Gabon; 2 École doctorale régionale d’Afrique centrale en Infectiologie tropicale, Franceville, Gabon; 3 Centre Hospitalier Régional George RAWIRI, Lambaréné, Gabon; 4 Department of Parasitology, Leiden University Medical Center (LUMC), Leiden, The Netherlands; 5 Fondation pour la Recherche Scientifique (FORS), Cotonou, Benin; 6 Department of Emerging Infectious Diseases, Institute of Tropical Medicine (NEKKEN), Nagasaki University, Nagasaki, Japan; 7 Division of Biomedical Science, Institute of Medicine, University of Tsukuba, Tsukuba, Japan; 8 Institute of Tropical Medicine, Vietnam Research Station, Nagasaki University, Nagasaki, Japan; 9 Division of Infectious Diseases and Tropical Medicine, Department of Medicine 1, Medical University of Vienna, Vienna, Austria; 10 National Research Center for the Control and Prevention of Infectious Diseases (CCPID), Nagasaki University, Nagasaki, Japan; 11 Graduate School of Biomedical Sciences, Nagasaki University, Nagasaki, Japan; 12 Institut für Tropenmedizin, Universität Tübingen, and German Center for Infection Research (DZIF), Tübingen, Germany

**Keywords:** Dengue, Serotypes, Circulation, Moyen Ogooué, Gabon, Symptoms

## Abstract

**Background:**

Despite dengue virus (DENV) outbreak in Gabon a decade ago, less is known on the potential circulation of DENV serotypes in the country. Previous studies conducted in some areas of the country, are limited to hospital-based surveys which reported the presence of some cases of serotype 2 and 3 seven years ago and more recently the serotype 1. As further investigation, we extend the survey to the community of Moyen Ogooué region with the aim to assess the presence of the dengue virus serotypes, additionally to characterize chikungunya (CHIKV) infection and describe the symptomatology associated with infections.

**Method:**

A cross-sectional survey was conducted from April 2020 to March 2021. The study included participants of both sexes and any age one year and above, with fever or history of fever in the past seven days until blood collection. Eligible volunteers were clinically examined, and blood sample was collected for the detection of DENV and CHIKV using RT-qPCR. Positive samples were selected for the target sequencing.

**Results:**

A total of 579 volunteers were included. Their mean age (SD) was 20 (20) years with 55% of them being female. Four cases of DENV infection were diagnosed giving a prevalence of 0.7% (95%CI: 0.2–1.8) in our cohort while no case of CHIKV was detected. The common symptoms and signs presented by the DENV cases included fatigue, arthralgia myalgia, cough, and loss of appetite. DENV-1was the only virus detected by RT-qPCR.

**Conclusion:**

Our results confirm the presence of active dengue infection in the region, particularly DENV-1, and could suggest the decline of DENV-2 and DENV-3. Continuous surveillance remains paramount to comprehensively describe the extent of dengue serotypes distribution in the Moyen-Ogooué region of Gabon.

## Background

Dengue virus (DENV) infections are the most widely spread arboviral infections worldwide [1–5]. They are endemic in tropical regions where more than 400 million people are exposed each year [6, 7]. Dengue fever (DF) is known to be an uncomplicated disease. However, a rapid change to hemorrhagic dengue fever (DHF) can be observed in individuals exposed to more than one of the four known serotypes; DENV-1; DENV-2; DENV-3; and DENV-4 serotype [8], raising the interest to the secondary heterologous infections in endemic countries [9].

In Africa, cases of infections due to the DENV were reported in many countries [2, 10–12] with non-homogenous distribution of the four genotypes. Indeed, dengue outbreaks were reported in the continent over the past decade, from 2011 to 2021 [4]. In that period, East Africa was the most affected [4] with DENV-2 serotype reported in Ethiopia from 2013 to 2018 [13], in Tanzania from 2014 to 2019 [14], while DENV-3 serotype was detected in Djibouti in 2014 [15]. Moreover, all four dengue serotypes were detected in Kenya between 2013 and 2018 [16, 17]. During the same period, three West African countries were affected by dengue outbreaks with Burkina-Faso reporting the presence of serotypes 1, 2, and 3 in 2013 and then from 2016 to 2017 [18, 19]. In central Africa, Angola and Cameroon reported outbreaks of the serotype 1 in 2013 [20] and 2017 [21], respectively. More recently in 2021, serotype 4 was detected for the first time in febrile patients in Yaoundé(Cameroon) [22]. However, Gabon experienced two concomitant outbreaks of dengue and chikungunya occurring in 2007 and 2010 in which the serotype 2 was reported [23–27]. This increases the risk of severe forms of the disease because of Antibody-dependent enhancement (ADE) can occur after infection [9]. Even unevenly distributed, the above information provides evidence of the presence of the four serotypes in the continent, increasing the risk of severe forms of the disease among populations and therefore ADE. ADE is a phenomenon in which antibodies that are produced during an initial infection with DENV, can enhance the entry of the same virus into host cells during subsequent infections. This enhancement can lead to more severe illness or increased infectivity. ADE can occur when non-neutralizing or sub-neutralizing antibodies, which are antibodies that do not effectively neutralize the virus, instead facilitate the entry of the virus into target cells by binding to it. This can happen because the antibodies attach to the virus in a way that allows it to bind more efficiently to receptors on host cells, promoting viral entry and replication [28, 29]. Since this epidemic period in Gabon, no other outbreak has been reported. However, the presence of serotype 2 and 3 was reported during epidemiological surveys conducted in 2016 and 2017 among febrile individuals in Lambaréné, Gabon [2, 30, 31], indicating a circulation of dengue virus in the population. Recently in 2021, two cases of DENV-1 and one case of chikungunya virus (CHIKV) were reported during a study aiming to assess the cause of fever in hospital-based patients in Lambaréné [32], indicating the presence of serotypes 1, 2, and 3 in the country. However, less is known about the distribution of dengue serotype in the population and the risk ofADE and severity of the disease. As further investigation, the present survey extends the investigation in the community with the aim to assess the presence of the dengue virus serotypes, as well as to characterize CHIKV infection in the local population, and describe the symptomatology associated with dengue virus infection in a Moyen Ogooué province of Gabon.

## Materials and methods

### Study design

This study was a cross-sectional survey conducted from April 2020 to March 2021, where febrile patients were recruited either among those visiting health facilities in the study area, or actively in the community including rural and urban areas.

### Study area

The study was conducted in the Moyen-Ogooué, one of the nine provinces of Gabon, located in the center of the country. The Moyen-Ogooué province consists of two departments:: The Ogooué et Lacs with Lambaréné (urban area) as the city capital located by road around 240 km from Libreville, the administrative capital of the country; and Abanga Bigne department with Ndjolé (semi-urban area) as the city capital and considered as the second most populated town in the region. Lambaréné and Ndjolé host all administrative structures, including four hospitals equipped with all medical services, several dispensaries, services units, trades, high schools, police stations, modern water, and electricity supply facilities. Lambaréné and Ndjolé are surrounded by several villages (rural areas) with limited health care infrastructures. A 2015 census report indicates that approximately 75% of the 69,287 inhabitants of the region live in urban areas [33]. The Moyen-Ogooué is known to be endemic for DENV and CHIKV [23, 24, 30, 31], where a surveillance system for viral diseases with epidemic potential has been established between Institute of Tropical Medicine of the Nagasaki university and the Centre de Recherches Médicales de Lambaréné, a medical research center located in Lambaréné city [34]where the study was conducted.

### Study population

Female and male volunteers of any age, beginning from one year old, who reside in the study area and have fever (axillary body temperature equal to or above 37.5 °C) or a history of fever within the past seven days from the day of inclusion, and who have sought medical care either at a hospital or within the community, were invited to participate in the surveyys.

### Sample size consideration

Our study was based on the monitoring of dengue virus infections and the characterization of its serotypes in the population. We therefore included all cases of fever or history of fever encountered in the community or in selected hospitals of the region during the study period.

### Study procedure and sample collection

A standardized questionnaire aiming to investigate the presence of clinical symptoms on the examination day or over the past seven days was administed to all eligible participants. Symptoms were recorded and classified in two groups: dengue related symptoms and other symptoms. Subsequently, a 4 ml venous blood sample was collected into a S-Monovette dry tube for the diagnosis of DENV and CHIKV infections. For later, we performed a Rapid Diagnostic Test (RDT) to diagnose malaria infection. Participants seen in the community, with fever at the time of the visit were invited to our research facility at CERMEL to be examined by a study physician. Participants tested positive for malaria were treated according to the national guidelines. For any other diagnostic, a medical prescription was provided to the participant or, if necessary, the participant was referred to an appropriate health care center for a further diagnosis.

### Laboratory analysis

All laboratory tests, including molecular detection and sequencing, have been performed at the Immunology and Molecular biology Laboratory of the Centre de Recherches Médicales de Lambaréné (CERMEL). 4 ml venous blood for serum collection was centrifuge at 3000 rpm for 10 min. The collected serum was stored at -80 °C for later usage.

### Viral RNA extraction

A total of 140 µl of stored serum was used for viral RNA extraction using the QIAamp RNA mini kit from QIAGEN® (Qiagen, Hilden, Germany) and following the manufacturer’s recommendations. The RNA extracts were then stored at -80 °C for long time.

### Reverse transcription quantitative polymerase chain reaction (RT-qPCR)

RT-qPCR for the detection of DENV-1, DENV-2, DENV-3, DENV4 and CHIKVV was performed in a 20 µl reaction using a One Step PrimeScript RT-qPCR Mix (2X) (TAKARA), which contains TaKaRa *Taq* HS enzyme. Each reaction mixture contained 10 µl of One Step PrimeScript RT-qPCR Mix, 2 µl of Specific Primer-Probe Mix (PPM), 0.4 µl ROX Reference Dye (50X), and 5.6 µl of H2O RNase-free (PCR grade) added to 2 µl RNA template. RT-qPCR assays were conducted using a Light Cycler 480 instrument (Roche, Basel, Switzerland) under the following conditions: 5 min at 52 °C, 1 min at 95 °C; and 45 cycles of 5s at 95 °C, and 60s at 60 °C. The primers and probes were designed using the sequences reported previously by Santiago [35]. DENV-1 (FAM-CTC YCC RAG ACG ACT TCA A-BHQ1, Fwd. CAA AAG GAA GTC GYG CAA TA, Rev CTG AGT GAA TTC TCT CTG CTR AAC), DENV-2 (FAM-CTC YCC RAG ACG ACT TCA A-BHQ1, Fwd. CAG GCT ATG GCA CYG TCA CGA T, Rev CCA TYT GCA GCA RCA CCA TCT C), DENV-3 (FAM-ACC TGG ATG TAG GAG CTT G-BHQ1, Fwd. GGA CTR GAC ACA CGC ACC CA, Rev CAT GTC TCT ACC TTC TCG ACT TG YCT), DENV4 (FAM-TYC CTA CYC CCG CAT TCC G-BHQ1, Fwd. TTG TCC TAA TGA TGC TRG TCG; Rev TCC ACC YGA GAC TCC TTC CA). CHIKV (FAM**-**AAC ATC TGC ACY CAA GTG TAC CAC AAAA GT**-**MGBEQ, Fwd CAG TGC GGC TTC TTC AAT ATG, Rev CGC ATT TTG CCT TCG TAA TG) Data from the RT-qPCR assays were analyzed using software included in the Light Cycler 480 system. RT-qPCR assays were performed in duplicate and samples showing cycle threshold (Ct) values under 40 were set as positive.

### Envelope gene sequencing for genotyping of detected DENV strains

The Amplification of the envelope gene of DENV-1 strain detected was performed using primers designed for two regions R1 (Fwd. CCGATTCAAGATGTCCAACA, Rev -CTTCCACCAATGTGGCTTCT) amplifying one region of 1071pb and R2 (Fwd. AACCACCTTTTGGTGAGACC, Rev GGTTGCTTCGAACATTTTTCC) amplifying a region of 858 pb of Gabon reference strain MG877557-DENV-1-Gabon2012-Env. The analysis was performed with the PrimeScript II High Fidelity One Step RT-PCR Kit (Takara Bio, Shiga, apan) in a reactional mix of 20 µl containing 10 µl of 2x One Step High Fidelity Buffer, 0.4 µl of PrimeScript II RT Enzyme Mix, 1.6 µl of PrimeSTAR GXL, 2 µl of Primer Mix (5 μm), 4 µl of RNase-free water and 2 µl of DENV-1 RNA in the following cycling program: 45 °C for 15 min; 94 °C at 2 min; 30 cycles 98 °C for 10 s; 60 °C for 15s; 68 °C for 1 min; 68 °C for 1 min before an agarose gel purification was performed with a QIAquick Gel Extraction Kit (Qiagen). For the sequence of reactions, PCR products were processed using the Big Dye Terminator v3.1 Cycle Sequencing Kit (Thermo Fisher Scientific, Waltham, MA, USA) and analyzed using an ABI3500 capillary sequencer (Thermo Fisher Scientific) to obtain sequence data. The serotypes of DENV strains were determined through BLAST analysis of the sequence data (https://blast.ncbi.nlm.nih.gov/Blast.cgi).

### Phylogenetic analysis

For phylogeny inference of the full-length envelope gene sequences of DENV-1 strains, a Bayesian analysis was performed with timestamped reference sequences, which included complete envelope gene sequences of African DENV-1 strains using Mega software V.4.0 software (https://www.megasoftware.net).

### Statistical consideration

Data collected were entered into Microsoft Excel 2016 and exported into GraphPad- prism 7 version 4.0 (GraphPad Software, San Diego, CA, USA) for analysis. Statistical analysis was mainly descriptive. Continuous variables were summarized as mean and standard deviation (SD) while categorical variables were summarized as proportion and 95% confidence interval (CI).

### Ethics consideration

The study protocol was reviewed by the Scientific Review Committee (SRC) of CERMEL (SRC N°2019-10) and was approved by the Institutional Ethics Committee of CERMEL under the number CEI-008/2019 and the National Ethics Committee of Gabon (CNE-Gabon) under the number PROT N°0085/2019/PR/SG/CNER, respectively. The study procedure, risks and benefits were explained to each participant and those who agree to participate provided a signed informed consent before any study procedure was performed. For illiterate adults, the informed consent process was conducted in the presence of a family (impartial) witness, and both adults signed the consent forms. For children younger than 18 years of age, the signed informed consent was provided by their parents or legal representative. The study was conducted according to the Good Clinical Practice principles of the international conference of harmonization and the declaration of Helsinki requirements.

## Results

### Study population characteristics

A total of 579 febrile patients were included in the study with a mean (± SD) age of 20 years old (± 20). Of these, as presented in Tables 1, 319 (55%) were female, given a 1.2 female-to-male sex-ratio. One out of two study participants 306 (53%) came from Lambaréné, the urban area, while 125 (22%) and 148 (26%) of their counterparts came from the semi-urban (Ndjolé) and rural areas, respectively.

**Table 1:** Characteristics of 579 participants included

	*n*	%
**Gender**		
Female	319	55.0
Male	260	45.0
Female to male sex-ratio	1.23	-
**Age (n, mean, SD)**	(510, 19.9, ± 19.5)	
Age group*		
1–10	227	39.2
11–20	96	16.6
21–40	113	19.5
41–60	49	8.5
> 60	29	5.0
**Location**		
Ndjolé (semi-urban area)	148	25.6
Lambaréné (urban area)	306	52.8
Rural areas	125	21.6

*Missing data on age: 65

### Clinical characteristics of the study population

As presented in Tables 2, 392 (68%) of the participants were enrolled at the health centers while the remaining 32% were seen in the community. At the time of inclusion, 488 (84%) participants had fever, and the other 91 (16%) reported history of fever in the past seven days. The body temperature of our study participants ranged from 36.0 °C to 40.5 °C. A total of 14 different clinical symptoms were recorded. Fatigue (53%, 95%, CI: 49–57), headache (45%, 95%, CI: 44–52), and loss of appetite (48%, 95%, CI: 41–50) represented the main recorded clinical symptoms. Vomiting (33%, 95%, CI: 30–37), abdominal pain (29%, 85%, CI: 25–33), caught (29%, 95%, CI: 25–32), and arthralgia (29%, 95%, CI: 24–32) were less represented. A total of 250 (48%) out of the 579 participants with fever had malaria.

**Table 2:** Clinical symptoms and malaria infection among participants

	*n*	%	95% CI (%)
**Site of inclusion**			
Community	187	32.3	-
Hospital	392	67.7	-
**Fever**			
Yes	488	84.3	
History of fever	91	15.7	
**Temperature (mean, SD)**	(37.65 °C, ± 0.86)		
**Clinical symptoms**			
Fatigue	308	53.2	49.1–57.3
Loss of appetite	278	48.0	43.9–52.1
Headache	263	45.4	41.3–49.9
Vomiting	193	33.3	29.9–37.1
Abdominal pain	168	29.0	25.3–32.7
Cough	167	28.8	25.1–32.5
Arthralgia	162	28.0	24.3–31.6
Diarrhea	96	16.6	13.5–19.6
Myalgia	81	14.0	11.1–16.8
Ear pain	78	13.5	10.7–16.3
Difficulty breathing	62	10.7	8.2–13.2
Constipation	51	8.8	6.5–11.1
Skin rash	20	3.9	2.2–5.5
Bleeding (epistasis)	17	2.9	1.55–4.31
**Concomitant malaria**	250	43.1	39.1–47.2

### Virus detection

All 579 participants were tested for DENV and CHIKV using RT-PCR. Four participants (0.7%; 95%CI: 0.02–1.8) were positive for DENV serotype 1 (DENV-1) while no case of CHIKV virus was found.

### Characteristics and distribution of symptoms among participants with dengue virus infection

Table 3 presents the characteristics of the four participants (two females and two males) positive for DENV-1 and which are 9, 18, 24, and 26 years old,. Among them three recruited in the community and the youngest in the hospital.All of them have their residence in Lambaréné. Regarding the reported symptoms, the two oldest participants had fever at the time of inclusion, while the two others reported history of fever. All of them presented at least five symptoms; with at least four are known to be related to DENV infection. These include fatigue (4 cases), myalgia (3 cases), arthralgia (3 cases) and ear pain (2 cases). For the participant aged 24-year-old, three dengue symptoms were reported: ear pain, breathing difficulties breathing, and an epistasis. None of them reported abdominal pain, but all presented coughed and had a loss of appetite as associated symptoms. None of the four participants positive for DENV were malaria positive.

**Table 3:** Characteristics and distribution of clinical symptoms among the four participants positive for dengue virus

	Participant 1	Participant 2	Participant 3	Participant 4
Serotype	DENV-1	DENV-1	DENV-1	DENV-1
Age in years	18	24	26	9
Sex	Male	Male	Female	Female
Location	Lambaréné	Lambaréné	Lambaréné	Lambaréné
Recruitment site	Community	Community	Community	Hospital
History of fever	Yes	No	No	Yes
Axillary temperature (°C)	37.1	37.5	38.1	36.8
Clinical symptoms	6	6	5	6
**Dengue infection Symptoms**				
Skin rash	No	No	No	No
Headache	No	No	No	Yes
Myalgia	Yes	No	Yes	Yes
Arthralgia	Yes	No	Yes	Yes
Vomiting	No	No	No	Yes
Ear pain	Yes	Yes	No	No
Difficulty breathing	No	Yes	No	No
Bleeding (epistasis)	No	Yes	No	No
Abdominal pain	No	No	No	No
Fatigue	Yes	Yes	Yes	Yes
**Other symptoms**				
Cough	Yes	Yes	Yes	Yes
Loss of appetite	Yes	Yes	Yes	No
Diarrhea	No	No	No	No
Concomitant malaria	No	No	No	No

### Phylogenetic analysis

**Fig. 1:**
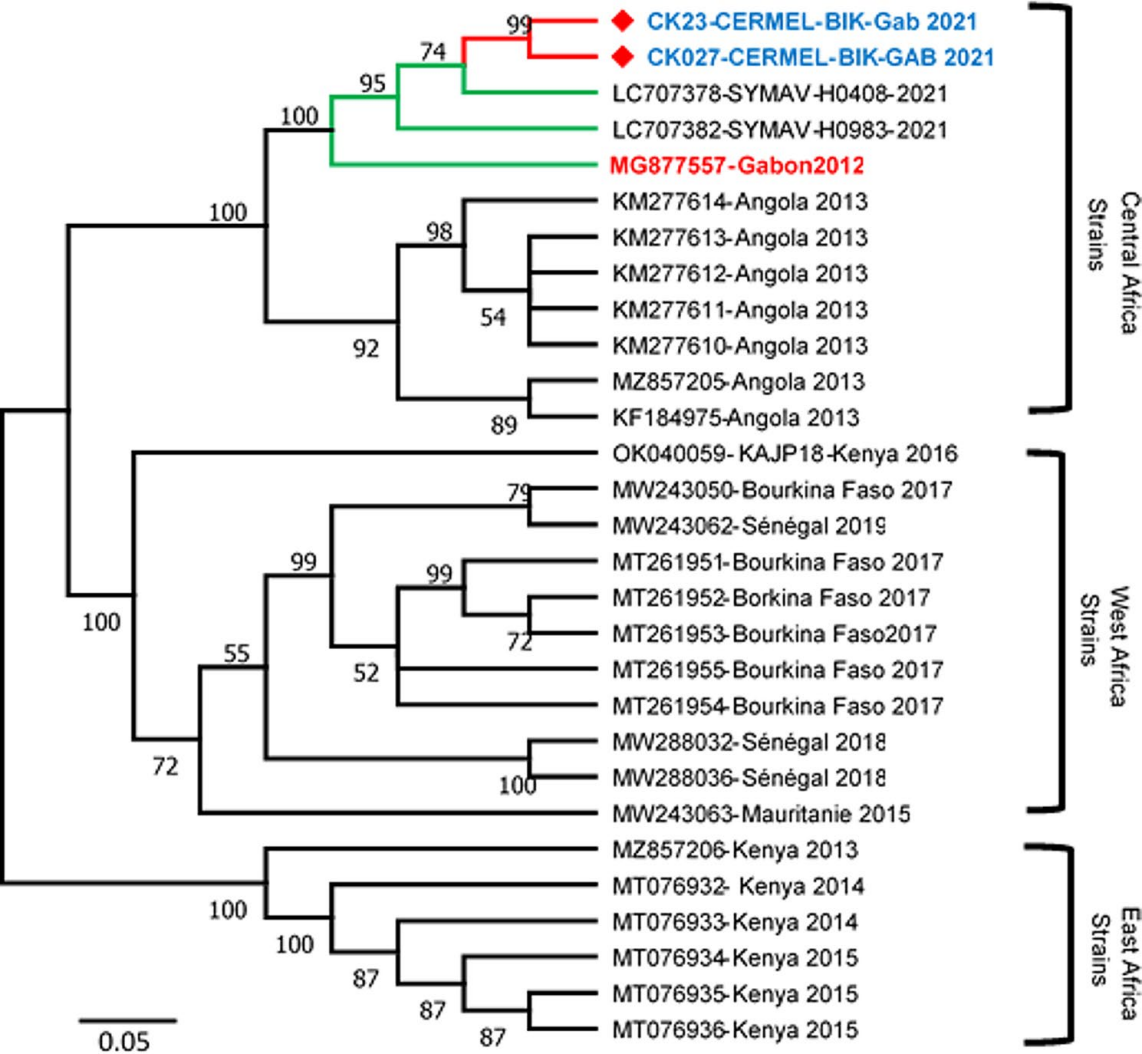
Phylogenetic analysis of partial envelope of dengue serotype 1 (1929 bp)

From all four positive samples, partial envelope glycoprotein gene was sequenced to investigate the genetic characteristics of DENV-1 strains. Two of these positive samples were detected with Ct values above 37. The low yield from genetic material of theses samples have not permitted to get exploitable sequences after sequencing run. Only the two samples CK023 and Ck027 with Ct value 29 and 32 respectively provided good sequences. Figure 1 represents the phylogenetic tree build only with Africans strains characterized during the last decade. The result highlights that the two sequences CK023-CERMEL-BIK-Gabon-2021 and CK027-CERMEL-BIK-2021, registered in Genbank with accession numbers OR135730 and OR135731 respectively, are similar to two strains LC707378 and LC707382 characterized in Lambaréné by Yuri in 2021. These strains belong to a clade in which Gabon strain/2012 is the common ancestor.

A maximum-likelihood tree was inferred with 1,000 bootstrap replicates. Bootstrap values of ≥ 70% are shown at the main nodes. Virus genotypes and lineages are shown on the right. The Gabonese strains detected in this study are shown in bold and blue.

## Discussion

The aim of the present study was to extend the surveillance of dengue virus serotypes and chikungunya virus in the community by expanding the collection of samples while maintaining routine screening of febrile patients presenting at the hospitals. Our results confirm the presence of dengue infection among hospital patients, and report for the first-time cases of dengue in the community but do not reveal ongoing cases of chikungunya in the study area.

The number of cases of dengue infection could be under or overestimated when surveys are conducted only in health facilities. Indeed, of the four DENV infection cases we found, three of them were found in the community and just one in a hospital setting, giving the evidence of DENV infection in the region. Previous studies reported cases of dengue virus infection from patients recruited in hospitals only [2, 30–32]. However, it is known that especially adults do not always go to health center for consultation in case of fever for several social reasons, as well as time and long distances but instead undergo the auto-medication using antipyretic and or antimalarial drugs with favorable outcome. And because of low severity of the infection [36]. This is particularly true in our region where fever is frequent and generally link to different infectious diseases as malaria, respiratory tract infection, bacterial infection [37], and mostly viral infection as hepatitis B and C [38]. Our results therefore emphasize the need to establish a surveillance based on the two settings, including community and hospital-based approaches to have the full picture of dengue infection in our target population.

The dengue virus infection remains active in Lambaréné region even if there is trend of decrease over time. We found a 0.7% (95%CI: 0.3–1.8) prevalence of dengue infection in our study population. This prevalence tend to be lower than the 2% (95%CI: 1.2–3.3) and 1.7% (95%CI: 1.1–2.8) reported respectively by Lim et al. in 2015 [31] and Abe et al. in 2017 [30] from febrile patients seen in Lambaréné hospital. Although the difference we observed between our finding and the previous reports is not clearly statistically significant, the trend could indicate a possible decrease in infection incidence in the local population, probably due to the appearance or disappear of dominant serotypes [23, 30, 31], thus reinforcing the idea of implementing surveillance of these viruses and their different genetic forms.

We provided additional evidence of the presence of dengue serotype 1 in our community, and failed to find the other serotypes; 2, 3, and 4 and therefore no risk of Antibody-dependent Enhancement (ADE). Our results could indicate the re-emergence of the serotype 1 in the local population after more than ten years, and a decline of the serotypes 2 and 3 in the region. Indeed, in one hand, the serotype 1 was reported for the first time in Lambaréné during the inter-epidemic period of 2009 with five cases, and in in 2010 with six cases [23]. The other hand, the serotype 2 has been reported in the two outbreak waves from 2007 to 2010 with 386 cases [23, 24], up to 2016 with two cases [31] while the serotype 3 was reported for the first time in 2010 with one case, and then in 2014 and 2017 with 17 cases in Lambaréné region [23, 30]. Indeed, as we did in both the community and hospitals, Yuri et al. reported DENV serotype 1 and none of the other serotypes and this study provides therefore more evidence for that. The dynamic of serotypes over time could indicate constant changes in the ecology of this arbovirus, above all with the temperature increasing which could differently affect the incidence of the dengue serotypes [39].

Analysis inferred of the phylogenetic tree constructed from the DENV1 sequences obtained indicates the presence of the same DENV-1 strain in Gabon population since 2012. The sequences of DENV-1 strains obtained in this study, are still those previously reported in 2020 by Yuri et al. [32]. The origin of the phylum indicates that the DENV-1 Gabon 2012 strain from the 2007–2010 epidemic period is their main origin [23]. So, the DENV-1 strain has been maintained over time, and based on the last seroprevalences reported showing a high exposition rate of the population to arboviruses in the region [2], we hypothesize existing genetic or environmental factors maintaining the circulation and transmission of the strain over time in a lower intensity. Indeed, as summarized by Lim et al. the transmission of dengue fever is related to several risk factors, including dengue virus’ presence and vitality, mosquitoes’ vectorial behavior and capacity, climate or weather conditions, human immunity, and activity [31]. In our region, such investigations need to be conducted to have the full picture of DENV in our target population [40].

Despite a high seroprevalences of CHIKV infection (61%) based on IgG antibodies detection reported in 2016 by Yuri et al. among patients recruited from hospitals in the region [2], we found no active cases of chikungunya in our study population. The diagnosis of active case of CHIKV infection remains rare in our community. Indeed, except during the epidemic period of 2010 which enable a molecular characterization of the virus in the country [24], only one case of active CHIKV infection has been reported by Yuri and collaborator in 2021 [32]. As IgG antibodies to ChikV remain detectable for years, we could assume a very low incidence of CHIKV infection in our community and the high seroprevalence observed could therefore be a result of cumulative cases over years. In addition to the low incidence of CHIKV infection we hypothesized, the absence of active cases we reported which opposes the high seroprevalence of IgG antibodies could also support the hypothesis that CHIKV circulates in the population inducing light clinical manifestation which did not required medical consultation, making difficult to catch active cases. Increase or reduce levels of several biomarkers such as cytokines or coagulation factor (factor VII) have been associated with the severity of CHIKV infection [41]. In our study area co-endemic with helminth infections known to deeply modulate the host immune system to prevent immune-mediated worm ejection [42], we can hypothesize a possible interaction between the two infections to explain the low clinical expression of CHIKV infections we observed. Therefore, to have full picture of chikungunya in our community, a community survey remains a important tool.

We were not able to characterize the clinical profile of dengue and chikungunya infection in our study population. The low number of positives cases for dengue virus did not permit to establish a specific dengue infection profile as Nkoghe et al. did in 2010 [26, 27]. Indeed, among 53 patients in Franceville, Nkoghe et al. were able to identify a minimum of nine common symptoms [26]. Only three dengue symptoms were common in our positive patients: fatigue, myalgia, and arthralgia. However, it is well known that these signs are not specific to dengue infection and can also be found in other disease such as malaria or respiratory tract infections which are also frequent in this area [43].

Surprisingly, we found no malaria-dengue virus co-infection despite that about half of the study population were infected with *Plasmodium* spp. On the four dengue cases, none was positive for malaria. Our finding is similar to those already reported by Fernandes et al., in 2016 in Lambaréné where an infection rate of 52% was found in hospital patients with fever and no case of arboviruses was found [37], probably because the study was conducted in hospital and among febrile children aged less than 15. However, our finding opposes the result of Nkenfou et al. who found a malaria-dengue virus co-infection rate of 19.5% in Cameroon [44]. As indicated by Gebremariam et al. on malaria and acute dengue virus coinfection in Africa [45], where dengue infection overlaps with malaria, the risk of co-infections is more important. From our side, we hypothesize that the very low prevalence of dengue infection observed explain the absence of co-infection with malaria.

In our study, we did not used the serological methods such as Enzyme Link ImmunoSorbent Assay (ELIZA) to detect IgM (recent infections). This could be presented as a limitation as the seroprevalences may provide the information on the exposure level of population to virus. We indeed focused on active cases. However, serological methods are sometime compromise by cross-reactions due to other pathogens in the same family and do not allow to assess the viral activity. Molecular and genetic methods used in the study allow to detect active infection and give a best virus characterization. The present study focused on Dengue and Chikungunya, the most prevalent arboviruses in the study area. For that reason, but also because of limited resources in our project, we have not extended our investigation to others arboviruses.

## Conclusion

Our results indicate the presence of the DENV-1 in the Moyen-Ogooué province, and especially in Lambaréné area. Although we reported a very low infection rate in our target community, our results indicate a necessity to reinforce the genomic surveillance of dengue virus. Community-based surveillance coupled with hospital-based surveillance could therefore be a relevant tool to roll-out the surveillance. Particularly for the early detection of outbreaks and characterization of occurrence of severe diseases related to emergence of DENV-4 or re-emergence of other serotypes 2, and 3 and therefore the risk of Antibody-dependent Enhancement (ADE), as our study provide additional evidence of the presence of serotype 1 in the region.

## Data Availability

No datasets were generated or analysed during the current study.
